# Genome Assembly of the Canadian two-row Malting Barley cultivar AAC Synergy

**DOI:** 10.1093/g3journal/jkab031

**Published:** 2021-02-04

**Authors:** Wayne Xu, James R Tucker, Wubishet A Bekele, Frank M You, Yong-Bi Fu, Raja Khanal, Zhen Yao, Jaswinder Singh, Brian Boyle, Aaron D Beattie, François Belzile, Martin Mascher, Nicholas A Tinker, Ana Badea

**Affiliations:** 1 Morden Research and Development Centre, Agriculture and Agri-Food Canada, 101 Route 100 Morden, MB R6M 1Y5, Canada; 2 Brandon Research and Development Centre, Agriculture and Agri-Food Canada, 2701 Grand Valley Road, Brandon, MB R7A 5Y3, Canada; 3 Ottawa Research and Development Centre, Agriculture and Agri-Food Canada, 960 Carling Avenue, Ottawa, ON K1A 0C6, Canada; 4 Plant Gene Resources of Canada, Saskatoon Research and Development Centre, Agriculture and Agri-Food Canada, Saskatoon, SK S7N 0X2, Canada; 5 Plant Science Department, McGill University, 21111 Lakeshore Road, Ste. Anne de Bellevue, Quebec, QC H9X 3V9, Canada; 6 Institut de Biologie Intégrative et des Systèmes, Université Laval, Québec, QC G1V 0A6, Canada; 7 Crop Development Centre, University of Saskatchewan, Saskatoon, SK S7N 5A8, Canada; 8 Département de phytologie, Université Laval, Québec, QC G1V 0A6, Canada; 9 Leibniz Institute of Plant Genetics and Crop Plant Research (IPK), Gatersleben, 06466 Seeland, Germany; 10 German Centre for Integrative Biodiversity Research (iDiv) Halle-Jena-Leipzig, 04103 Leipzig, Germany

**Keywords:** barley, reference genome, assembly, AAC Synergy

## Abstract

Barley (*Hordeum vulgare* L.) is one of the most important global crops. The six-row barley cultivar Morex reference genome has been used by the barley research community worldwide. However, this reference genome can have limitations when used for genomic and genetic diversity analysis studies, gene discovery, and marker development when working in two-row germplasm that is more common to Canadian barley. Here we assembled, for the first time, the genome sequence of a Canadian two-row malting barley, cultivar AAC Synergy. We applied deep Illumina paired-end reads, long mate-pair reads, PacBio sequences, 10X chromium linked read libraries, and chromosome conformation capture sequencing (Hi-C) to generate a contiguous assembly. The genome assembled from super-scaffolds had a size of 4.85 Gb, N50 of 2.32 Mb, and an estimated 93.9% of complete genes from a plant database (BUSCO, benchmarking universal single-copy orthologous genes). After removal of small scaffolds (< 300 Kb), the assembly was arranged into pseudomolecules of 4.14 Gb in size with seven chromosomes plus unanchored scaffolds. The completeness and annotation of the assembly were assessed by comparing it with the updated version of six-row Morex and recently released two-row Golden Promise genome assemblies.

## Introduction

Barley (*Hordeum vulgare* L.) is one of the most important crop species in the world. By acreage, it is the fourth largest crop in Canada and in the world (FAOSTAT 2020, accessed on 25 Jan 2021). In Canada, approximately 70% of the barley crop is used for feed and about 21% for malt (Tricase*et al.* 2018). The use of barley as a food has also been increasing since health claims have been approved by the US Food and Drug Administration (2006), European Food Safety Authority (EFSA) (2011), and Health Canada (2012) (Badea*et al.* 2018).

Barley is a diploid (2n = 2× = 14) inbreeding species with a large haploid genome of 5.1 gigabases (Gb) (Mayer *et al.* 2012). Since the late 1920s, barley has been widely used to study and utilize induced genetic variability (Schulte *et al.* 2009). Numerous barley genetic and genomic data were accumulated during the early 21st century, but the exploitation of these data was hindered by the lack of a complete genome reference. The International Barley Genome Sequencing Consortium (IBSC) was initiated in 2006 (Schulte *et al.* 2009) and reported the first barley reference genome in 2012 (Mayer *et al.* 2012). This IBSC V1 was constructed by shotgun sequencing of bacterial artificial chromosome (BAC) clones from the six-row barley cultivar (cv.) Morex with partial genome representation anchored to physical and genetic maps. Since then, both genetic research and crop improvement in barley have benefited from this partly ordered draft sequence assembly. In 2017, IBSC improved the first assembly by producing a highly contiguous reference genome sequence for barley (IBSC V2) based on Illumina-sequenced BACs combined with optical mapping and Hi-C chromosome conformation (Mascher*et al.* 2017). The IBSC V2 constitutes a core and an important community resource for cereal genetics and genomics.

Beyond the core barley reference genome, a barley pan-genomics project was proposed to capture the intraspecific diversity in genome content and structure in whole barley germplasm groups through the assembly of high-quality genome sequences from a large number of accessions (Monat *et al.* 2019a). For individual genomes, whole-genome shotgun assemblies for four domesticated two-row barley genotypes: Barke, Bowman, Haruna Nijo, and Igri (Mayer *et al.* 2012) and one wild accession B1K-04-12 (Hübner*et al.* 2009) have been published by IBSC. Subsequently, several draft genome assemblies were constructed based on deep-coverage Illumina sequencing: the Japanese malting barley Haruna Nijo (Sato *et al.* 2016), two Tibetan hulless barleys qingkecvs. Lasa Goumang (Zeng et al. 2015) and Zangqing320 (Dai *et al.* 2018), and a wild barley (*Hordeum spontaneum*) (Liu *et al.* 2020). To reduce the cost per assembled genome Monat *et al.* (2019b) developed a computational pipeline (TRITEX) for chromosome-scale sequence assembly of wheat and barley genomes and constructed an improved annotated reference genome assembly for barley cv. Morex (Morex V2). Using the same TRITEX pipeline, Schreiber *et al.* (2020) recently released the genome of a European two-row barley cultivar, Golden Promise.

AAC Synergy has a different parentage from Golden Promise and is a hulled two-row spring malting barley cultivar developed at the Agriculture and Agri-Food Canada (AAFC) Brandon Research and Development Centre, Brandon, MB (Legge *et al.* 2014). With a combination of high yield, good foliar disease resistance and malting quality, AAC Synergy is currently the second most commonly sown two-row malting barley cultivar in Western Canada (McMillan *et al.* 2020). Due to its consistent malting attributes, it has been recognized and added in 2015 to the recommended list of malting varieties on both sides of the border, in Canada by Canadian Malting Barley Technical Centre (CMBTC) and in United States of America by American Malting Barley Association (AMBA) and continues to be part of these lists to date (CMBTC 2015 and 2021; Gribbins 2015; AMBA 2020). The AAC Synergy reference genome would represent a useful addition to the genomic resources for genomic and genetic studies of malting barley and benefit research specific to Canadian barley malting quality and disease resistance.

## Materials and methods

### Plants and DNA extraction

Approximately 80 AAC Synergy seeds were sown in four 20-cm diameter pots containing soilless Pro-Mix. Plants were grown on a single cart in a growth chamber under 18°C 18 h light and 15°C 6 h dark. At the two-leaf stage, leaves from all plants were cut from the shoot with scissors and submersed in 2 L of liquid nitrogen for 10 minutes. The tissue was held in a freezer at –80°C until required.

High molecular weight (HMW) DNA was isolated by Bio S&T Inc. (St-Laurent, QC, Canada). DNA quality was evaluated via agarose gel electrophoresis where the majority of DNA fragments were sized between 50 and 250 Kb. The DNA was sent to the Plateformed’AnalysesGénomiques of the Institut de BiologieIntégrative et des Systèmes (Université Laval, Québec City, QC, Canada) for DNA library preparation.

For PacBio sequencing, 1–2 grams of barley leaf samples after being lyophilized, were mailed to the DNA Sequencing Center, Brigham Young University, for DNA extraction and library construction.

### Library preparation and sequencing

The PE450 libraries were prepared by fragmenting 500 ng of HMW DNA using a Covaris M220 for 80 s. The sheared DNA was size selected on a 2% agarose gel cassette using a BluePippin (SAGE Science) set to elute between 62 and 74 minutes. Libraries were prepared with 25 ng of the 400–600bp fraction using the NEBNextUltraII kit following the manufacturer’s instructions. Individual libraries were barcoded using Illumina TruSeq HT barcodes. Final libraries were quantified using a Qubit fluorometer and controlled for quality using a BioAnalyzer DNA high sensitivity chip. An equimolar amount of library was pooled with other libraries for sequencing. The PE450 libraries were sequenced on an Illumina NovaSeq SP PE 250 format (2 × 250 bp reads) at the Centre de Services et d’ExpertisesGénome Québec (Montréal, QC, Canada). Multiple runs of the pooled libraries achieved an approximate 70× coverage.

Mate-pair libraries (MP9) of 8–10 Kb were prepared with a Nextera mate-pair kit using the gel plus protocol. The size selection was performed on a BluePippin 0.75% agarose gel cassette in pulsed-field mode using broad range elution settings set at 9 Kb. Individual libraries were barcoded and sequenced on an Illumina Novaseq S4 PE 150 format (2 × 150 bp reads) at the Centre de Services et d’ExpertisesGénome Québec (Montréal, QC, Canada).

The 10X chromium libraries were produced as per the 10X Genome Chromium library protocol v1 (10X Genomics) with size selection (> 48.5 Kb). Two individual libraries were prepared and uniquely indexed for multiplexing, and quantified by qPCR (Kapa Biosystems, Wilmington, MA, USA). These libraries were sequenced on the Novaseq S4 PE 150 format (2× 150 bp reads).

For PacBio sequencing, DNA was extracted at the DNA Sequencing Center, Brigham Young University. The DNA was sheared to 60 Kb and size selected on a BluePippin to collect fragments exceeding 35 Kb. The PacBio CLR library was constructed using the SMRTbell^™^ Template Preparation protocol. This library was sequenced on a single 8M SMRT cell of Sequel II system for 15 h.

The chromosome conformation capture sequencing (Hi-C) data of barley cv. Golden Promise Hi-C (SRR8922888) were downloaded from the NCBI sequence read archive (Bioproject PRJNA533066; Schreiber *et al.* 2020).

### Illumina contig assembling

TRITEX pipeline scripts (Monat *et al.* 2019b) (https://tritexassembly.bitbucket.io/) were used to fulfill the assembly processes. Overlapping single reads of the PE450 libraries were merged with BBMerge(Bushnell *et al.* 2017). The base errors in merged PE450 reads were corrected by BFC (Li 2015). Minia3 (Chikhi *et al.* 2016) was used to assemble corrected and trimmed PE450 reads into contigs. TRITEX applied iterative Minia3 runs with increasing k-mer sizes (100, 200, 300, 350, 400, 450, and 500). In the subsequent runs, the input reads as well as the assembly of the previous iteration were used as input for the assembler.

### PacBio contig assembling

The PacBio long sequence reads were assembled into contigs by first performing backbone assembly and then error correction (File S1). First, the uncorrected PacBio reads were all-vs-all overlapped by Minimap2 (Li 2018). Then the overlaps were further assembled into graphs using Miniasm(Li 2016). These graph based contigs were finally corrected using PE450 reads by Recon (v1.4.13) (Vaser*et al.* 2017). A total of three rounds of corrections were performed for the final contigs.

### Contig merging

The PE450 contigs and PacBio contigs were merged using the quickmerge program (Chakraborty *et al.* 2016). First, the two contig assemblies were aligned with the Nucmer program. Then, repeats and duplicates were filtered out using delta-filter of 10 Kb. Last, quickmerge took PE450 contigs as query and PacBio contigs as reference for merging.

### Mate-pair scaffolding and gap filling

Nextera junction adapters and short-insert contaminants were removed from mate-pair reads using NxTrim(O’Connell *et al.* 2015). Mate-pair reads were corrected with BFC using the hash table of k-mer counts generated from the PE450 reads. SOAPdenovo2 (Luo *et al.* 2012) was used to scaffold the error-corrected MP9 reads. The best N50 of scaffolds was selected by testing a range of parameters for “pair_num_cutoff” (minimum of read pairs linking two sequences) for each library. The internal gaps of the scaffolds were filled by the error-corrected PE450 reads using GapCloser(Luo *et al.* 2012).

### Super-scaffolding by 10X chromium sequences

Edges between scaffolds were defined only if they were supported by molecules from more than one Chromium library. In the initial graph, POPSEQ marker sequences (the WGS contigs of the International Barley Sequencing Consortium 2012) were aligned to the scaffolds using Minimap2. Branches were resolved by applying heuristics to obtain subgraphs. The mean position of molecules linking to other scaffolds was used to determine the orientation of a scaffold within a super-scaffold.

### Chromosome conformation

To generate a full chromosome assembly, we utilized the Hi-C data for Golden Promise (SRR8922888, Schreiber *et al.* 2020) which carried the native chromatin folding to increase the contiguity to full chromosome size. Hi-C links scaffolds to super-scaffolds. Super-scaffolds were ordered and oriented as described by Beier *et al* (2016). Intra-chromosomal Hi-C matrices were visually inspected in a locally installed R Shiny app (Mascher *et al.* 2017).

### Base complexity coverage analysis

We used the spectra-cn function from the K-mer Analysis Toolkit (KAT v2.3.1) (Mapleson *et al.* 2017) to check for base content inclusion in the contigs and the scaffolds. KAT first generated a k-mer frequency from reads. It then identified how many times k-mers from each part of the distribution appeared in the assembly being compared. The spectra-cn function generated plots for the k-mer frequency distribution and sequencing errors from the contigs.

### Gene content analysis

Two gene databases were used to assess how many genes were contained in assemblies or assembling steps. The first approach was done with BUSCO (Benchmarking Universal Single-Copy Orthologs, v4.0.6) and embryophyta_odb10 (November 20, 2019) (Simão *et al.* 2015). It assessed the completeness of a genome by identifying conserved single-copy, orthologous genes. The second approach used a FL-cDNA dataset (HvuFLcDNA_rep.fa, 2013/02/18) which consisted of 22,651 sequences generated from the cultivar Haruna Nijo (Sato *et al.* 2009). These sequences were created from 12 different conditions and representing a good snapshot of the barley transcriptome. The 22,651 FL-cDNAs were mapped to the AAC Synergy pseudomolecule using Gmap with the following parameters: a minimum identity of 98% and a minimum trimmed coverage of 95%. Both databases were used to identify the number of gene sequences existed in both the AAC Synergy pseudomolecule and the databases which would give an impression on the segmentation of the pseudomolecule, highlighted by cDNAs which were split within or across chromosomes, and encompassed a complete or a partial gene sequence.

### Gene annotation

Transcript annotation was conducted by both knowledgebase and *ab initio* prediction. The assembly was searched against the barley reference transcriptome dataset (BaRT v1.0) (Rapazote-Flores *et al.* 2019) using Gmap (version 2018-03-25) (Wu and Watanabe 2005) with the following parameters: -f 2 -n 1–min-trimmed-coverage = 0.8–min-identity = 0.9. All BaRT transcripts were aligned to the assembly and the output was converted into GFF format. The August program (version 3.3.3) (Stanke *et al.* 2008) was used for *ab initio* gene prediction of the AAC Synergy assembly based on the wheat model (Hoff and Stank 2018).

### Repeat annotation

The final assembly was analyzed for repetitive regions using RepeatMasker (version 4.1.0) (Smit *et al.* 2013–2015) with the TREP Repeat library (trep-db_complete_Rel-19) (Wicker *et al.* 2002). The output of RepeatMasker was condensed using the perl script “onecode-to-find-them-all” with the parameters–strict and–unknown (Bailly-Bechet *et al.* 2014).

### Comparative analysis

The AAC Synergy scaffolds were compared to the Morex V2 (https://doi.org/10.5447/IPK/2019/8) and Golden Promise V1 assembly (GCA_902500625) pseudomolecules, using Minimap2 for assembly-to-assembly alignment. Alignment records were written to PAF format and imported into R for visualization and calculation of summary statistics. At sequence base level, the row-type gene *VRS1* was compared among the three cultivars by BLAST alignment. The *VRS1* sequence (1568 bp) of a two-row barley (*Hordeum vulgare* subsp. *vulgare*) was downloaded from the genbank (AB259782.1) (Komatsuda *et al.* 2007).

## Results

### Genome assembly

From two PE450 libraries, approximately 742 million 2 × 250bp paired reads were generated, providing an estimated 74× coverage of the genome (Table 1). One PacBio SMRTbell adapted DNA library generated a total of 6,679,274 continuous long reads (CLR) with a read length N50 of 27 Kb and an approximate 22× coverage depth. These two data sets constituted the base composition of the assembly. As shown in Table 2, the contigs formed by short reads in the PE450 libraries alone had a N50 of 40 Kb and an assembly size of 4.03 Gb. The PacBio backbone contigs after PE450 polishing increased the contig size (4.12 Gb), but did not significantly increase the assembly size. The merged assembly represented a size of 4.81 Gb.

**Table 1:** Read statistics and coverage

Name	Library type (number)	Insert size	Read length	No of read pairs#	Coverage depth
PE450	PCR-free paired-end (2)	400–470 bp	2 × 250 bp	742,581,027	74×
PacBio	CLR SMRT bell adapted DNA library (1)	28 Kb (N50)	27 Kb (N50)	6,679,274	22×
MP9	Nextera mate-pair (2)	6–9 Kb	2 × 150 bp	683,249,642	30×
10X	10X Chromium (2)	> 48.5 Kb	2 × 150 bp	883,458,414	30×
Hi-C	TCC(1)(Schreibe*et al* 2020)	–	2 × 100 bp	232,874,136	–

**Table 2:** Assembly statistics, size, and coverage

	N50	Number	Length >10Kb	#Number >1Mb	Largest	Size
Contigs						
PE450	40.1Kb	246,874	3.47 Gb	0	447.9Kb	4.03 Gb
PacBio	428.7Kb	15,752	4.12 Gb	386	3,021.9Kb	4.12 Gb
Merged	453.8Kb	184,671	4.26 Gb	636	4,290.9Kb	4.81 Gb
Scaffolds						
MP9	570.8Kb	154,804	4.38 Gb	888	4,354.6Kb	4.85 Gb
10X	456.8Kb	223,485	4.25 Gb	709	4,101.0Kb	4.85 Gb
Chr conformation						
Hi-C	2,321,7Kb	216,027	4.25 Gb	1040	18,379.5Kb	4.85 Gb
Pseudomolecule	537,338.4Kb	8	4.14 Gb	8	546,689.9Kb	4.14 Gb

The MP9 scaffolding increased the N50. However, since most of the PacBio reads had an insert size similar to that of the 10X chromium library insert size, the 10X chromium iteration did not show any significant N50 improvement. We integrated the downloaded barley cv. Golden Promise Hi-C data which carried the native chromatin folding to increase the contiguity to full chromosome size. Further removing of the scaffolds that were less than 300 Kb resulted in a final assembly of 4.14 Gb and seven chromosomes plus an extra chromosome containing the unassigned scaffolds.

### Assembly completeness in base complexity content

**Figure 1:**
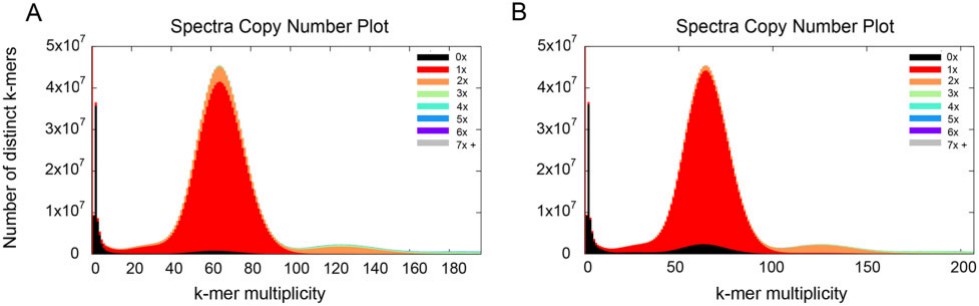
Spectra cn plots comparing k-mers from the PE450 reads to k-mers in (A) the merged contig assembly and (B) the pseudomolecule assembly. Y-axis represents the number of distinct k-mers from PE450 reads. X-axis is the k-mer coverage on genome size. 0×, 1×, 2×,… represent the copy number of k-mers found in the assemblies.

A good assembly should contain all base complexity (k-mers) of the original genomic DNA. It is assumed that the PE450 reads with high coverage have sampled every part of the underlying genome, though stretches of identical repeats are omitted from the assembly. Ideally, an assembly should contain all k-mers found in the reads (not including k-mers arising from sequencing errors) and assure that no k-mers are absent from the reads. As shown in the spectra-cn plot in Figure 1, A–B, the majority of the k-mers generated from PE450 reads with a coverage of ∼60× were represented in the contig assembly and the pseudomolecule assembly in the merged orange and red peak indicating the assemblies captured almost all genome DNA source sequence reads that had k-mers of coverage depth >5×, particularly those k-mers that had 60× coverage depth.

Only the k-mers that had a low coverage depth (<5×) and very few high-depth k-mers were not found in the contigs and the pseudomolecule assemblies (shown in black), which indicates either sequencing errors or assembly errors. The black region under the main peak was very small, indicating that most of this content from the reads was present in the assembly. The content that appeared to the right of the main peak and was present two or three times in the assembly represents repeats.

Both assemblies contained a fraction of k-mers with a depth of 0 in the PE450 reads (red bars along the Y axis), which reflects k-mers that appeared in the assemblies but did not appear in the PE450 reads, indicating the presence of miss-assemblies. After the removal of scaffolds less than 300 Kb in size, the number of missing PE450-derived k-mers (0× coverage) from the assemblies was greater in the pseudomolecule assembly (black peak at around 60 multiplicity on X-axis in Figure 1B)compared to the contig assembly (black peak in Figure 1A). In addition, the number of single-copy k-mers found in the assembly(red peak) with ∼60× coverage in the PE450 reads was also increased, when compared to two-copy k-mers (orange peak) in the contig assembly.

### Assembly completeness in terms of gene content

**Figure 2:**
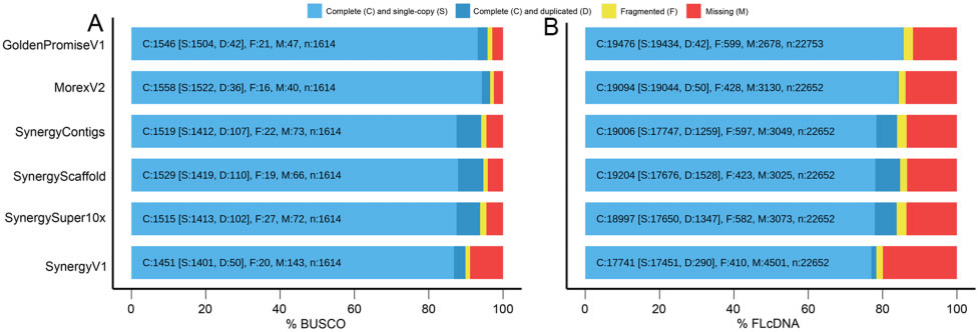
Completeness assessment of the AAC Synergy assembly in comparison to the previous steps of the assembly process and the published barley references MorexV2 and Golden Promise V1 for both (A)the BUSCO analysis and(B)the FL-cDNA mapping analysis.

Among the total 1614 genes in the BUSCO database (v4.0.6), the AAC Synergy contigs included 1519 (94.1%) complete genes. The single copy genes represented 87.5% of all database genes, which was lower than the pseudomolecule assemblies of published Golden Promise V1 (93%) and Morex V2 (94%) (Figure 2A). This suggested that the long sequence reads used in our assembly could retain a larger number of original repeat sequence segments (multi-copy k-mers) in the assembly which may have been collapsed in the short read assemblies, *e.g.*, the Golden Promise V1 and Morex V2. Throughout the assembly process, only a slight improvement occurred in scaffolding via MP9 (94.7%). As the total size of the pseudomolecule assembly decreased by removal of scaffolds of less than 300 Kb in size, some genes were likely removed from the pseudomolecule assembly.

The second analysis (based on the FL-cDNA database) showed similar results (Figure 2B). The contig assembly already contained 84% complete genes in comparison to the 86% and 84% of the Golden Promise V1 and MorexV2, respectively. The final assembly contained 78.3% complete single-copy genes. Similar to the BUSCO analysis, the number of duplicated complete genes and the number of fragmented genes decreased in the AAC Synergy assembly, when compared to the previous Contig, Scaffold, and Super-scaffold assembly steps.

### Assembly transcript annotation

Through a BaRT dataset search using Gmap, a total of 46,845 unique transcripts were discovered on seven chromosomes and one undefined chromosome (Table 3). Gmap searched multi-exon cDNAs of a gene from BaRT against genome assembly. Gmap not only searched sequence homology (similar to BLAST), but it also generated accurate gene models and located splice sites. As shown in Table 3, the total number of genes discovered in AAC Synergy, though not done with *de novo* annotation, is slightly higher than that of Golden Promise V1 but lower than that of Morex V2. These differences may be partly biased by different genetic relationships with Haruna Nijo, so they cannot be used to definitively compare assembly quality. All three assemblies showed similar numbers of genes on each chromosome. However, the AAC Synergy V1 shows more continuity with a remarkably decreased number of gaps. The *ab initio* analysis predicted about 10-fold more gene models than the GMAP-based annotations. This is expected, since gene predictions often contain high numbers of false positives such as TEs and pseudogenes (Table S1). Future integration of RNA-seq data confirmation and gene functional definition by gene ontology (GO) could be helpful to characterize additional genes.

**Table 3:** AAC Synergy gene distribution on chromosomes

Chr	1H	2H	3H	4H	5H	6H	7H	ChrUn	Total
*Morex V2*									
len(Mb)^*^	508	657	612	609	582	557	617	68	4210
gap#	19750	26182	23635	21160	23579	21778	24330	27989	188403
gene#	7878	10263	9498	7398	9936	7956	9794	1268	63991
*GP V1*									
len(Mb)	463	597	557	560	525	502	561	237	4001
gap#	47227	61686	56858	52591	54157	50194	57692	201418	581823
gene#	5392	7074	6501	4879	6612	5311	6562	2823	45154
*AAC SynergyV1*									
len(Mb)	456	545	538	535	494	480	538	521	4108
gap#	2476	2839	2788	2686	3083	2581	3003	22305	41761
gene#	6229	7091	7356	5364	7457	5862	7239	6476	46845

* len(Mb) is sequence bases after removal of gaps. ChrUn: not mapped to any chromosomes

### Assembly of repetitive regions

The majority of the barley genome contains repetitive sequences. RepeatMasker analysis with the TREP repeat library identified 82.3% of the AAC Synergy assembly V1 as transposable elements (Table 4) with almost all belonging to the retrotransposon element class. This transposable element content was similar to Morex V2 (81.0%) and Golden Promise V1 (81.9%) when analyzed in the same manner. The representation of long terminal repeat (LTR) retrotransposon families was similar in these three assemblies (74.7%, 73.0%, and 72.3%). Interestingly, AAC Synergy assembly V1 had a slightly higher (2–3%) number of retro-elements, but a lower (2–3%) content of overall trans-elements than Golden Promise V1 and Morex V2 (Table 4).

**Table 4:** Identified repetitive elements in the AAC Synergy assembly

Category	AAC Synergy V1	Golden Promise V1	Morex V2
***Class I: Retrotransposon***			
LTR	74.7	73.0	72.3
LTR/Copia	23.4	22.2	23.3
LTR/Echo	0.00009	0.00011	0.00010
LTR/Gypsy	50.1	49.7	47.9
LTR/unknown	1.1	1.1	1.1
non-LTR			
LINE	1.0	1.0	1.0
SINE	0.077	0.081	0.080
***Class II: DNA Transposon***	8.5	8.8	8.7
DNA/CACTA	7.3	7.6	7.6
DNA/Harbinger	0.38	0.39	0.38
DNA/Mariner	0.26	0.27	0.26
DNA/Mutator	0.46	0.47	0.46
DNA/hAT	0.025	0.025	0.025
DNA/unknown	0.032	0.033	0.032
*Low-complexity*	0.062	0.055	0.053
*Simple-repeat*	0.71	0.57	0.53
*Unclassified*	0.007	0.007	0.007

* Values represent percentage coverage of the genome

### Comparison with Morex V2 and Golden Promise V1 assemblies

To assess the use of another source of Hi-C data and the assembly at the pseudomolecule level, we plotted alignments between chromosomal pseudomolecules of AAC Synergy V1 and Morex V2, Golden Promise V1 and inspected Hi-C contact matrices. The visual inspection of chromosomal alignments indicated a high concordance and a high collinearity of AAC Synergy V1 to either Morex V2 (Figure S1 A) or Golden Promise V1 (Figure S1 B). The contact matrices showed a typical diagonal line, indicating the scaffolds were contacted and connected by neighboring chromatin (Figure S1 C). No obvious mis-assemblies were present in the AAC Synergy V1 pseudomolecule. A slight Rabl pattern (Tiang*et al.* 2012) was seen in the contact matrices plot which shows the chromosomes with centromeres at one nuclear pole and telomeres at the other (the Rabl configuration). For example, the Rabl pattern at 250 mb of chromosome 3H (Figure S1 C).

The row-type gene *VRS1* was compared at sequence level. As expected, the complete sequence (1568 bp) of the *VRS1* from *Hordeum vulgare* subsp. *vulgare* was mapped on chromosome 2H (chr2H: 512462086-512460519) of the AAC Synergy assembly, being 100% identical alignment without mismatch nor gaps. The two-row Golden Promise assembly demonstrated the same identical alignment to this *VRS1* gene sequence on chr2H as the AAC Synergy assembly. However, the six-row barley Morex V2 exhibited 99.7% identical with three mismatches and one gap in alignment to this two-row *VRS1* genes on chr2H.

## Discussion and conclusion

In this study we combined deep short-read sequencing with shallow long-read sequencing to produce the first Canadian barley genome assembly. The resulting assembly had longer contiguousness and retained more repeat segments of the original genome than the previously reported barley Morex V2 and Golden Promise V1 assemblies. This work provides the barley research community with a Canadian two-row barley draft reference genome that will aid in the discovery genes responsible for the unique Canadian malting traits. However, due to the use of HiC data from an alternate two-row cultivar, Golden Promise, care should be taken in the interpretation of structural analysis.

Five rounds of contig assembly using Minia3 were recommended in the TRITEX pipeline (Monat *et al.* 2019b). However, the N50 of assembled unitigs was unexpectedly small in the first try. Examining the PE450 sequences showed a higher than expected degree of heterozygosity, probably because the genomic DNA was not from a single homozygous haplotype, but from mixture of 80 seeds. In future, this should be ensured by practicing single-seed descent and verified with genome-wide molecular markers (*e.g.*, GBS) at the stage of panel selection. Elevated levels of heterozygosity due to recent outcrossing would lead to complications in *de novo* sequence assembly, and are avoidable in an inbreeding crop (Monat *et al.* 2019a). For sequences containing residual heterozygosity, we ran Minia3 to assemble contigs. This algorithm uses heuristics (tip removal, bulges removal, and erroneous connections removal) to collapse homologous sequences and remove duplicated sequences. In this study, we tested the pooling of DNA from 80 individuals for conveniently collecting enough DNA material in limited time. The result suggested this is a feasible alternative method.

The basic units, contigs which are continuous without gaps, are crucial for a successful assembly. We expect a contig assembly with a N50 of >20 Kb and assembly size > 4.5 Gb for a barley assembly. Merging PacBio data increased the assembly size from 4.02to 4.81 Gb from the PE450 contigs. We found that these data sets of high-depth short reads and low-depth long reads worked well by the approach of the PacBio backbone contigs polished by PE450 reads. We also investigated other related hybrid methods. For example, we tried to assemble long reads that were first corrected by short reads. However, the low-depth long reads were dramatically trimmed during the correction (genome size of 1.6 Gb) and this resulted in an assembly of only 0.45 Gb. When we tried the approach of linking short-read contigs by long reads, the final assembly reached to 4.02 Gb. Although the MP9 and the 10X chromium data did not provide significant improvement in scaffolding or super-scaffolding (because the contigs merged with long sequence reads had an N50 of 453.8 K and most of the 9 K MP9 and ∼100 K 10X sequences fell within these contigs), larger basic contigs would result in a smaller number of gaps in the final assembly.

A limitation of the current study is that we did not generate Hi-C data from the same source of AAC Synergy but used Hi-C data from Golden Promise. However we make the follow assumptions: (1) the chromosome conformation between these two-row barleys was very similar; (2) the conformation was interpreted only for those Golden Promise Hi-C joins that aligned to AAC Synergy super-scaffolds; and (3) the cases with alignment but wrong conformation are likely to be rare. This is similar to the reference-guided approach that uses a closely related reference genome to quickly cluster, order, and orient genome assembly contigs into pseudomolecules (Alonge*et al.* 2019). Even though the scaffolds have been assigned to each chromosome, the orientation or arrangements of the scaffolds in some regions of chromosomes could be incorrect. Therefore, this assembly with long contiguousness can be used for sequence based variation studies, but it is not recommended for a structural variation analysis. The barley genome IBSC v1 with many mis-orientated regions had been improved to v2 with a great effort, although inversion errors could still exist. Our next study will be focused on the validation/correction of segment orientation of AAC Synergy V1 assembly. As we noted, a good large scale collinearity with several small scale inversions was observed between chromosomes of AAC Synergy and the other two barley assemblies. Once Hi-C data of AAC Synergy and other cultivars are available, we will be able to investigate these structural variations more thoroughly. In addition, the current V1 draft will be further improved in the following aspects: adding sequence data newly generated from a single homozygous haplotype of AAC Synergy will lift the overall homozygosity of assembled data, which is expected to increase the gene contents and completeness. Though the Hi-C data from one cultivar could be used for assembling multiple genomes, it is worth inspecting if the Hi-C data from the same cultivar will improve the assembly. For example, why the typical Rabl pattern (Tiang*et al.* 2012) was missing from the contact matrices plot using Golden Promise Hi-C data. The transcript annotation will be more comprehensive when the RNA-seq data will be available for the same cultivar AAC Synergy.

Here, we added a new barley genome assembly to the barley researcher community that is especially relevant for the Canadian two-row barley studies. This draft reference genome can be directly used for gene discovery in AAC Synergy and for investigations of barley genome, diversity investigation, and allows for more informative, comparative genomic analyses in barley.

## Supplementary Matreial

Supplementary material is available at *GENETICS* online.

## Data availability

Raw sequence data of the paired-end PE450, mate-pair MP9, 10X chromium, PacBio, and the AAC Synergy genome assembly fasta file were deposited under NCBI BioProject database (ID: PRJNA665698). Supplemental Material available at figshare: https://doi.org/10.25387/g3.13568381.
